# Spatiotemporal dynamics of net primary productivity and its influencing factors in the middle reaches of the Yellow River from 2000 to 2020

**DOI:** 10.3389/fpls.2023.1043807

**Published:** 2023-01-27

**Authors:** Wenxi Xuan, Liangyi Rao

**Affiliations:** ^1^ College of Soil and Water Conservation, Beijing Forestry University, Beijing, China; ^2^ Key Laboratory of State Forestry and Grassland Administration on Soil and Water Conservation, Beijing, China

**Keywords:** net primary productivity, climate change, MODIS, spatio-temporal variation, the middle reaches of the Yellow River

## Abstract

**Introduction:**

Net primary productivity (NPP) is an important indicator used to characterize the productivity of terrestrial ecosystems. The spatial distribution and dynamic change in NPP are closely related to regional climate, vegetation growth and human activities. Studying the spatiotemporal dynamics of NPP and its influencing factors plays a vital role in understanding ecosystem carbon sink capacity.

**Methods:**

Based on MODIS-NPP data, meteorological data, and land use data from 2000 to 2020, we analyzed the spatiotemporal variation characteristics and influencing factors of NPP in the middle reaches of the Yellow River (MRYR) by using unary linear regression analysis, third-order partial correlation analysis, and Sen+Mann-Kendall trend analysis.

**Results:**

The results showed that the annual average NPP of the MRYR was 319.24 gCm^-2^a^-1^ with a spatially decreasing trend from the southern part to the northern part. From 2000 to 2020, the annual average NPP experienced a fluctuating upward trend at a rate of 2.83 gCm^-2^a^-1^, and the area with a significant upward trend accounted for 87.68%. The NPP of different land use types differed greatly, in which forest had the greatest increase in NPP. Temperature had a negative correlation with NPP in most parts of the MRYR. Water vapor pressure promoted the accumulation of NPP in the northwestern MRYR. The areas with a positive correlation between NPP and water vapor pressure accounted for 87.6%, and 20.43% of the MRYR area passed the significance test of P< 0.05.

**Conclusion:**

The results of the study highlight the impact of climate factors and land-use changes on NPP and provide theoretical guidance for high-quality sustainable development in the MRYR.

## 1 Introduction

Vegetation can regulate the carbon cycle in the ecosystem through photosynthesis and respiration. A large amount of vegetation has been planted to regulate the amount of surface solar radiation, increase atmospheric humidity and adjust the local temperature, which promotes energy flow and material circulation in the ecosystem (Ji et al.,2020). The climate system provides suitable environmental conditions for vegetation growth. With continuous changes in temperature, the climate that affects plant growth has been widely studied by scholars (Gang et al., 2017; Sun et al., 2019). Net primary productivity (NPP) is the total amount of organic matter produced by the photosynthesis of vegetation minus autotrophic respiration consumption. As an important ecological indicator of the carbon budget of the terrestrial ecosystem (Potter et al., 2003; Piao et al., 2008), NPP not only reflects the photosynthetic production capacity of vegetation without human factors but also represents the quality of the terrestrial ecosystem and plays an active role in the surface carbon cycle (He and Zhang, 2006; Wang et al., 2021; Zhang et al., 2022). Therefore, the study of NPP is of great significance to the global carbon cycle and is conducive to the early realization of the “carbon peak” and “carbon neutrality” targets worldwide. In the mid-20th century, scholars at home and abroad began to pay extensive attention to the influence of climate change on NPP (Guo et al., 2012; Hossain et al., 2021). However, different climate factors have different mechanisms of influence on NPP in different regions (Zarei et al., 2021; Shen et al., 2022; Wergifosse et al., 2022). Solar radiation and annual average temperature are the main influencing factors of NPP change in the Guangxi karst area (Liu et al., 2017). Solar radiation and water vapor pressure were the main climatic factors affecting NPP in Hunan Province (Yan et al., 2022). In Hubei Province, NPP was positively related to temperature (Mao et al., 2022). In northern China, rainfall accelerated vegetation growth and promoted the accumulation of NPP, but temperature limited the increase in NPP (Ren et al., 2021). The effect of solar radiation and precipitation on NPP in Inner Mongolia was stronger than that of temperature (Hao et al., 2021). However, in different periods, the climate factors affecting NPP change in the same region were different. From 1982 to 1999, the enhancement of solar radiation was beneficial to the accumulation of NPP (Ke et al., 2003), while from 2000 to 2015, precipitation and temperature significantly promoted NPP increases in the Yangtze River Basin (Feng and Xie, 2022). From 1982 to 1999, NPP was mainly affected by solar radiation and temperature, while from 2000 to 2020, temperature and precipitation became the dominant climatic elements of NPP in the Qinghai-Tibet Plateau (Piao et al., 2006; Liu et al., 2022). Climate factors affect the physiological processes of vegetation by changing environmental conditions, thereby influencing the increase in NPP. However, vegetation growth is affected not only by climate factors but also by land use changes. For example, with the rapid urban expansion in Guangdong Province, a large amount of forest and cropland were occupied by construction land, which was not conducive to the accumulation of NPP (Jiang et al., 2016). From 2001 to 2015, the “Grain for Green” project was carried out in the Shule River Basin of Gansu Province and forest and grassland replaced a number of croplands, leading to a significant increase in NPP (Zhou et al., 2021). Because NPP is affected by many factors, NPP shows complex responses temporally and spatially. The driving mechanism of the spatial-temporal variation in NPP at the regional level remains unclear.

The middle reaches of the Yellow River (MRYR) flow through the Loess Plateau, which is located in humid, subhumid arid and semiarid zones with diverse vegetation types. Due to improper land use and excessive exploitation, the Loess Plateau has a large amount of sediment washing. The fragile ecological environment seriously limited the economic development of the surrounding provinces and cities. Therefore, strengthening the control of soil and water loss in the MRYR is an inevitable requirement to comply with the goal of high-quality development in the Yellow River Basin (Li et al., 2022). Planting a large amount of vegetation effectively alleviated the contradiction between water and sediment (Wang and Liu, 1999). Ecological measures such as “Grain for Green” and “Three North shelterbelts” have been implemented in the MRYR (Wei et al., 2018), resulting in a significant increase in vegetation coverage, which was directly reflected in the dynamic changes in NPP (Xin et al., 2007; Wang et al., 2009). Previous studies have mainly concentrated on the spatial and temporal distribution of NPP and its correlation with precipitation and temperature in the whole region of the Yellow River Basin (Wang et al., 2022; Xiao et al., 2022). Owing to the Yellow River basin flowing through a wide area, the complicated ecological environment and the obvious regional differences between the upper, middle, and lower reaches, it is difficult to study the NPP of the Yellow River basin as a whole to achieve accurate local implementation. Meanwhile, the MRYR suffers from serious soil and water losses, has become the main source of sediments and is the most fragile region in the Yellow River basin. The MRYR basin’s high-quality development is constrained by the conflict between water and sediment (Gao et al., 2011). Analyzing the variation in spatial and temporal characteristics of NPP in the MRYR is helpful for the evaluation of regional ecosystem productivity, which can directly reflect the change in the regional terrestrial carbon cycle and is an important index to reveal the regional carbon source/sink process. Clarifying the spatial-temporal variations in NPP and its response to climate factors is vital to establish scientific underpinnings for achieving “carbon peak” and “carbon neutral” and promoting green economic development in the MRYR.

Therefore, the MRYR was selected as the research object. Based on Moderate Resolution Imaging Spectroradiometer (MODIS) NPP data, meteorological data, and land use data from 2000 to 2020, we sought to describe trends in NPP and identify the main drivers of NPP. To answer this question, we proposed the following hypotheses: (1) the NPP significantly increased in the past 21 years in the MRYR; (2) the change in land use types may promote the accumulation of NPP in the study area; and (3) temperature was negatively correlated with NPP in most areas of the MRYR.

## 2 Materials and methods

### 2.1 Study area

**Figure 1:**
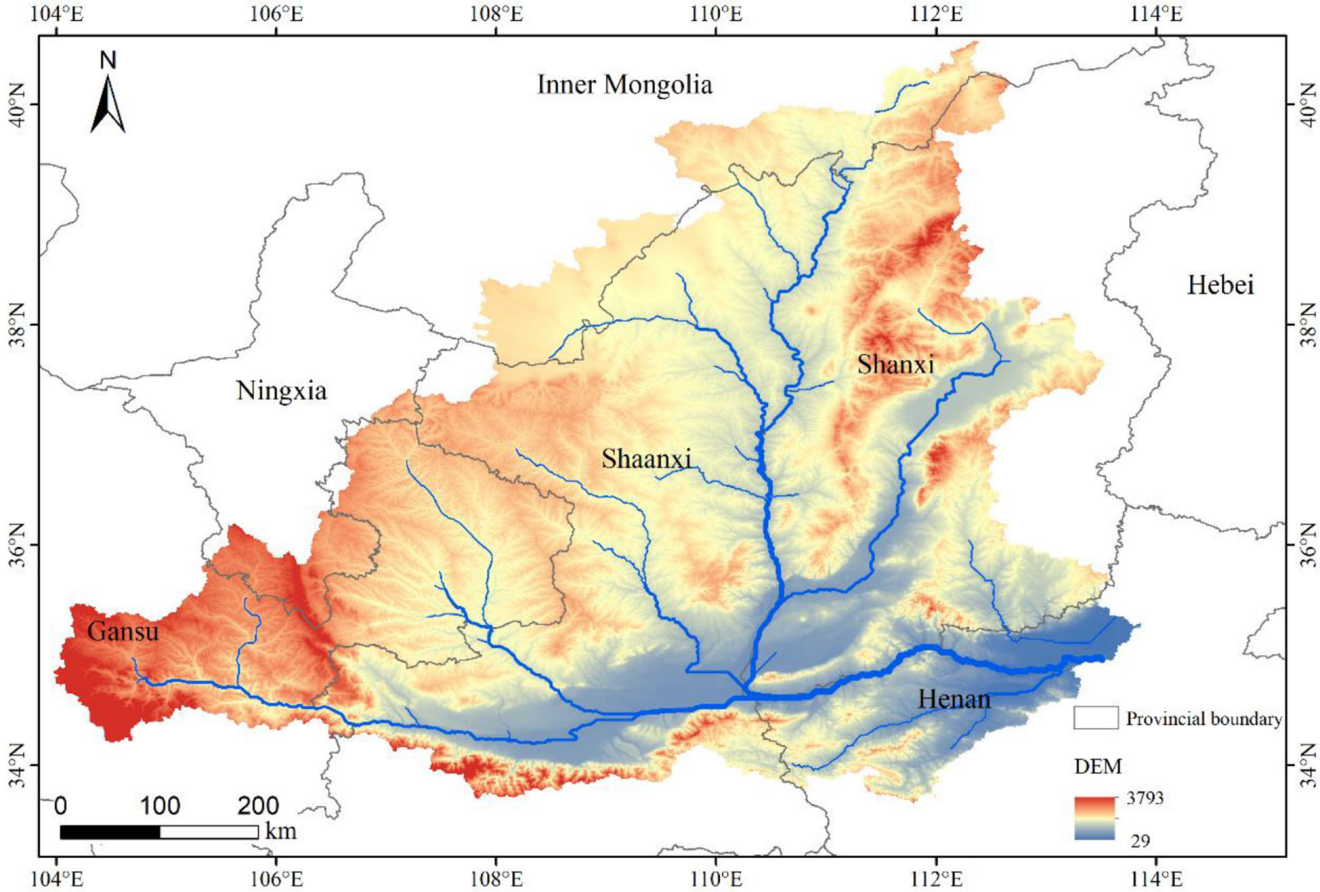
Location of the study area.

The MRYR refers to the area from the Toudaoguai hydrological station to the Huayuankou hydrological station, which is located at 32° ~42° N and 104° ~113° E (**Figure 1**) and crosses the six provinces of Shaanxi, Inner Mongolia, Shanxi, Henan, Gansu, and Ningxia. The MRYR reaches the Qinling Mountains in the south, the Lvliang Mountains in the east, the Loess Plateau in the middle and the Ordos Plateau in the northwest. The total length of this reach is approximately 1234.6 km, and the drainage area is approximately 362000 km^2^, accounting for 45.7% of the Yellow River Basin (He et al., 2022). The MRYR includes seven primary tributaries, such as the Kuye River, Tuwei River, and Wuding River, from north to south. The water source in the MRYR is mainly supplied by precipitation, which is mainly concentrated in summer. The precipitation in winter is low, with an average annual precipitation of 500–800 mm and an average annual temperature of 9.2–10.7 °C, indicating an arid to semiarid climate (Wang et al., 2021). Vegetation types in the MRYR mainly include sparse shrubby steppe, grassland belts, coniferous forest belts and deciduous broad-leaved forest belts (Yan et al., 2018). Grassland is widely distributed in the study area, accounting for more than 40% of the total area. The dominant species were *Artemisia ordosica*, *Leymus secalinus* and *Stipa bungeana* (Jiang et al., 2015). Cropland is mainly planted with wheat, corn and sweet potato, and is distributed in plain and hilly areas. The forests are mainly distributed in high mountains and middle mountains. The forest is mainly composed of secondary broad-leaved forest, mixed coniferous, broad-leaved forest and temperate coniferous forest. The tree species are *Betula platyphylla*, *Populus davidiana*, *Robinia pseudoacacia*, *Pinus tabulaeformis*, *Platycladus orientalis* and *Querusmongolica*. The main species of shrubs include *Hippophae rhamnoides* and *Caragana korshinskii* (Jian et al., 2022). In addition, the basin has poor corrosion resistance and serious erosion. Therefore, the situation of soil erosion in this area is very severe.

### 2.2 Data and processing

In this study, NPP data were derived from MOD17A3HGF of NASA (https://ladsweb.modaps.eosdis.nasa.gov/) from 2000 to 2020. The spatial resolution of the data was 500 m, the data format was GeoTIFF, and the projection format was WGS84. After the data were acquired, the MRT (MODIS Reprojection Tool) tool available on the NASA website was used for splicing, cutting and other operations. Then, the images processed by MRT were cropped by ArcGIS10.2, and the invalid values were removed. These data have undergone a series of rigorous technical processes with high accuracy and have been widely used by a large number of scholars (Ma et al., 2021; Gang et al., 2022; Ma et al., 2022). DEM (digital elevation model) data were derived from the geospatial data cloud (https://www.gscloud.cn/), and were mainly extracted from the boundary of the MRYR. The land use data were extracted according to the basin boundary from the Resource and Environmental Science and Data Center (https://www.resdc.cn/). For the convenience of research, the land use data were divided into six ground feature categories: cropland, forest, grassland, waterbody, construction land, and bare land. Bare land with low vegetation coverage mainly includes wild grassland, saline alkali land, marshland and sandy land. Meteorological data, including precipitation, temperature, water vapor pressure and solar radiation, were downloaded from the Google Earth Engine (GEE) remote sensing cloud platform. GEE is a geographic data analytics platform with a powerful climate database and high-performance computing services for fast data acquisition. As a remote sensing platform, its ability to quickly analyze data makes it an invaluable tool for studying global climate change (Patel et al., 2015; Ravanelli et al., 2018). Meteorological data were obtained from Terra Climate University of Idaho’s monthly climate and climate water balance dataset on global land surfaces (IDAHO_EPSCOR/TERRA CLIMATE). TerraClimate is a dataset of monthly climate and climate water balance on the global land surface and is currently the highest spatial resolution climate data product (Abatzoglou et al., 2018). This dataset provides the spatial distribution of climate factors by the climate-assisted interpolation method and water balance model (Patel et al., 2015; Ravanelli et al., 2018). Based on the vector boundary of the MRYR, the temperature, precipitation, solar radiation and water vapor pressure data from 2000 to 2020 were extracted year by year on the GEE cloud platform, and then the outliers were eliminated by ArcGIS. The partial correlation analysis was conducted with NPP data in MATLAB software.

### 2.3 Methodology

#### 2.3.1 Trend analysis of NPP

The univariate linear regression method was used to analyze the interannual change trend of NPP data (Zhao et al., 2018). The formula for trend analysis is expressed as follows:


(1) $$\begin{array}{c} Slope=\frac{n\sum_{i=1}^{n}i\times NPP_{i}-\sum_{i=1}^{n}i\sum_{i=1}^{n}NPP_{i}}{n\times \sum_{i=1}^{n}i^{2}-{\left(\sum_{i=1}^{n}i\right)}^{2}} \end{array}$$

where Slope is the interannual rate of NPP change, n is 21 for years from 2000 to 2020, and NPP_i_ is the amount of NPP in year i.

#### 2.3.2 Sen+Mann-Kendall trend analysis

The Theil-Sen Median method is also known as Sen slope estimation. The calculation method is efficient, and the error caused by observation has little influence on the result, so it is suitable for long time series data (Chen et al., 2022). The formula for the Sen+Mann-Kendall trend analysis is expressed as follows:


(2) $$\begin{array}{c} \beta =Median\left(\frac{NPP_{i}-NPP_{j}}{i-j}\right),\forall i>j \end{array}$$

where 1<j<i<n, n is the total time sequence length; NPP_i_ and NPP_j_ are the sample time series dataset; and Median is the median function. If β > 0, NPP showed an increasing trend; if β< 0, NPP showed a downward trend.

The Mann-Kendall (MK) test is used to test the change trend of variables over time, which does not require data to follow the normal distribution, and data loss and anomalies do not influence the results. This method is usually used for a significance test of the trend of long-term sequence data. The formula of test statistic S is expressed as follows:


(3) $$\begin{array}{c} S=\sum_{i=1}^{n-1}\sum_{j=i+1}^{n}sgn\left(NPP_{j}-NPP_{i}\right) \end{array}$$

where sgn() is the symbolic function. The formula is expressed as follows:


(4) $$\begin{array}{c} sgn\left(x_{j}-x_{i}\right)=\{\begin{array}{c} +1\;x_{j}-x_{i}>0\\ \;0\;\;x_{j}-x_{i}=0\\ \;-1\;x_{j}-x_{i}<0\; \end{array} \end{array}$$

The test statistic Z is used for the trend test, and the formula of the Z value is expressed as follows:


(5) $$\begin{array}{c} Z=\{\begin{array}{c} \frac{S}{\sqrt{Var\left(S\right)}}\;\;\left(S>0\right)\\ \;\;\;\;0\;\;\;\;\;\;\left(S=0\right)\\ \frac{S+1}{\sqrt{Var\left(S\right)}}\;\left(S<0\right) \end{array} \end{array}$$

where the formula of Var is expressed as follows:


(6) $$\begin{array}{c} Var\left(S\right)=\frac{n\left(n-1\right)\left(2n+5\right)}{18} \end{array}$$

When |Z|≤Z_1−α/2_, it accepts the null hypothesis, that is, there is no clear trend; if |Z| >Z_1−α/2_, it rejects the null hypothesis, namely, the trend of a significant change. Z_1−α/2_ is the corresponding value of the given significance level α = 0.05 in the standard normal distribution table, that is, Z_1−α/2_ = ± 1.96. When the absolute value of Z is greater than 1.96, it indicates that the trend has passed the significance test with 95% reliability.

#### 2.3.3 Partial correlation analysis

There are many factors affecting NPP. To analyze the correlation between only one variable and NPP, the influence of other variables needs to be removed. Therefore, this paper adopted third-order partial correlation analysis to achieve the correlation analysis of the three factors and used a T-test to test the results of partial correlation analysis (Tian et al., 2019). The formula of partial correlation analysis is expressed as follows:


(7) $$\begin{array}{c} r_{ij\cdot mnh}=\frac{r_{ij\cdot mn}-r_{ih\cdot mn}r_{jh\cdot mn}}{\sqrt{\left(1-r_{ih\cdot mn}^{2}\right)\left(1-r_{jh\cdot mn}^{2}\right)}} \end{array}$$

Where *r_ij·mn_*, *r_ij·mn_* and *r_jh·mn_* are the partial correlation coefficients of variables i, j, i, h and j, h, respectively. The formula of the T- test is expressed as follows:


(8) $$\begin{array}{c} t=\frac{\sqrt{n-k-2}\cdot r}{\sqrt{1-r^{2}}} \end{array}$$

where t is the statistic value of the T- test, r is the partial correlation coefficient of the corresponding variable, n is the number of samples, k is the number of controllable variables, and n-k-2 is the degrees of freedom.

## 3 Results

### 3.1 Interannual variation in NPP

**Figure 2:**
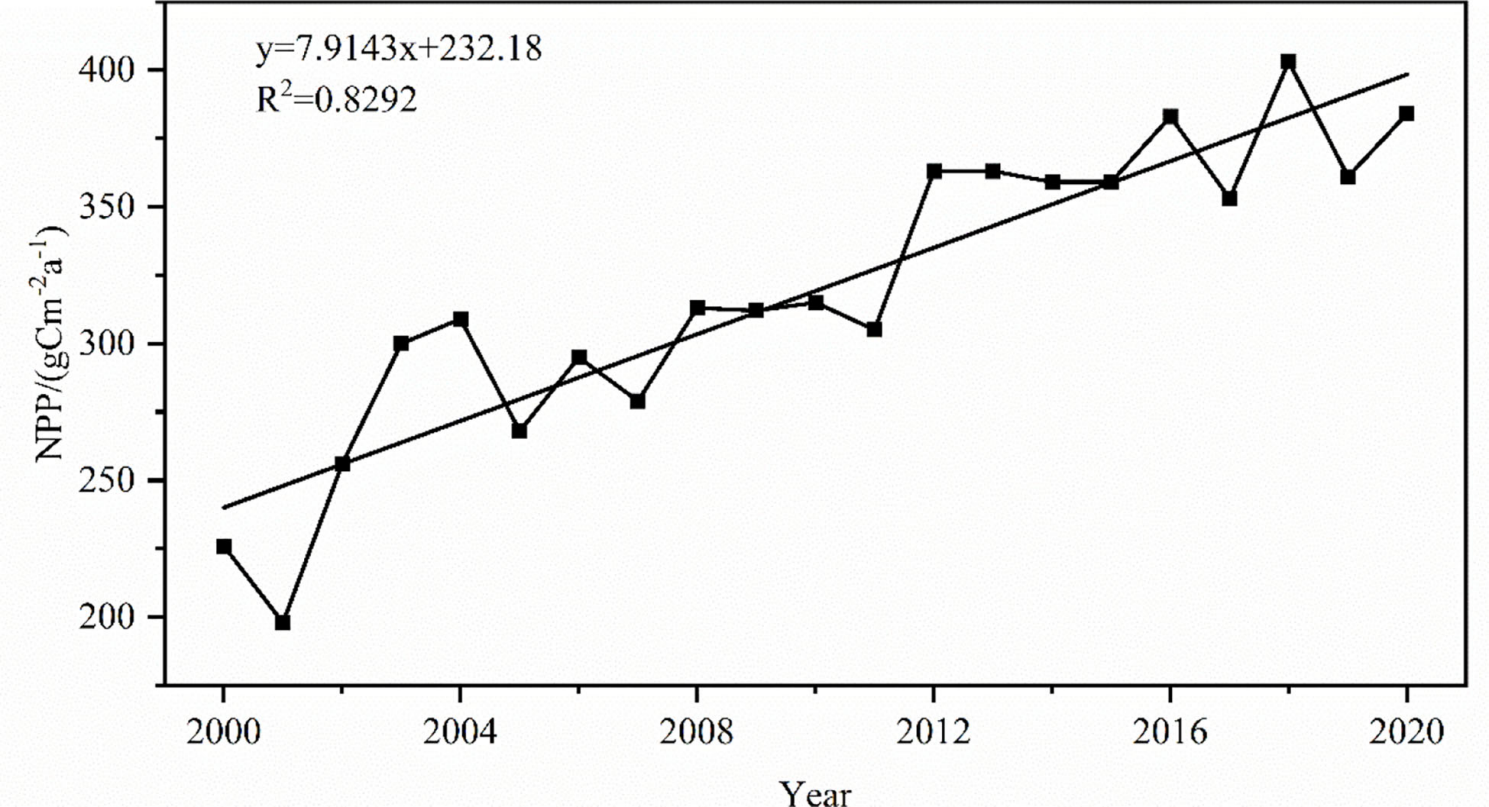
The changing trend of the annual average NPP from 2000 to 2020.

Based on the statistical analysis of NPP in the MRYR from 2000 to 2020, the annual average NPP was 319.24 gCm^-2^a^-1^. The annual variation in NPP ranged from 198.21 gCm^-2^a^-1^ to 403.57 gCm^-2^a^-1^, reaching a peak in 2018 and a low value in 2001. The annual average NPP of the MRYR showed a significant increasing trend (P< 0.01) (**Figure 2**).

### 3.2 Spatial distribution characteristics of NPP

**Figure 3:**
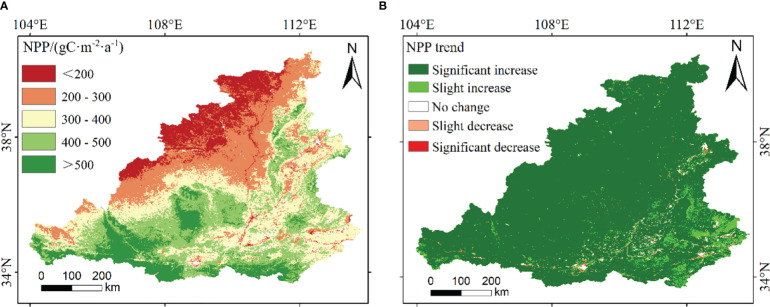
The spatial distribution **(A)** and significance level **(B)** of annual average NPP in the MRYR.

From 2000 to 2020, the spatial distribution of the annual average NPP in the MRYR was significantly different (**Figure 3A**), gradually decreasing from south to north. The NPPs in the Qinling Mountains and Ziwuling were higher, and those in the Mu Us Sandy Land were lower. The annual average NPP in the MRYR ranged from 52.65 to 1048.17 gCm^-2^a^-1^, of which 12% of the regional average NPP was within 200 gCm^-2^a^-1^, mainly distributed in southern Inner Mongolia, northwestern Shaanxi, and eastern Ningxia; 51% of the regional NPP average value was 200–400 gCm^-2^a^-1^, which was mainly distributed in northeastern Shaanxi and western Shanxi. The vegetation in central Shaanxi, southwestern Shanxi and eastern Gansu was dense, and the average value of NPP was as high as 400–600 gCm^-2^a^-1^. The maximum value occurred in the southern part of the MRYR in 2018, and the lowest value appeared in 2002 in southern Inner Mongolia. A total of 97.35% of the MRYR showed an increasing trend, of which the significantly increased area accounted for 87.68%; only 0.84% of the areas showed a downward trend, and 0.2% of the areas showed a significant downward trend (**Figure 3B**). At the spatial scale, NPP increased significantly in the western part of the study area. The decreasing area was mainly scattered in the southern part of the MRYR.

### 3.3 NPP variation characteristics in different land use types

**Figure 4:**
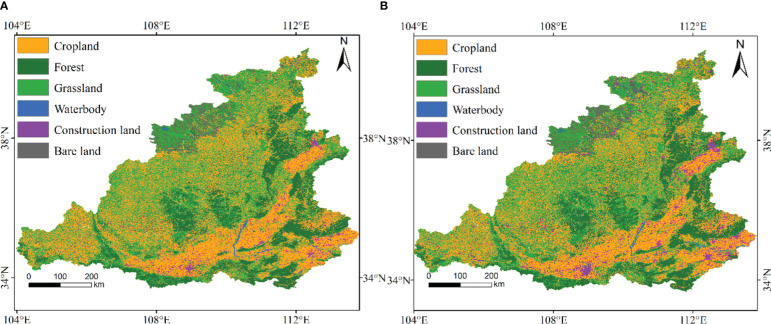
Spatial distribution of different land use types in the MRYR in 2000 **(A)** and 2020 **(B)**.

Based on the analysis of the land use transfer matrix in the MRYR from 2000 to 2020, the results showed that the land use types were mainly cropland, forest and grassland (**Table 1**). From 2000 to 2020, a total of 140305 km^2^ of land changed in land use mode, among which a large number of trees and grasses were planted, and part of the cropland was occupied. Therefore, the cropland was greatly reduced and was mainly converted to grassland (38439 km^2^, 27.5%) and forest (9552 km^2^, 6.8%). The conversion of cropland to forest resulted in an increase of 1.82 TgC in NPP (**Table 2**). The areas of grassland converted to cropland and forest were 34262 km^2^ (28.6%) and 12001 km^2^ (10.0%), respectively, which increased NPP by 10.49 TgC. Due to the rapid development of the city, a large amount of cropland was occupied by construction land, which increased the construction land by 8515 km^2^ (6.1%), resulting in a loss of 0.73 TgC in NPP. The transformation of other land use types resulted in a lower NPP transfer. At the spatial scale (**Figure 4**), the conversion of cropland into grassland was mainly distributed in northern Shaanxi and eastern Gansu. The NPP in this region increased significantly. The conversion of grassland into forest was mainly distributed in central Shaanxi and western Shanxi. In the areas where such land use changes occurred, the NPP showed a significant increasing trend. The overall area of grassland slightly increased, but the spatial distribution showed obvious changes.

**Table 1:** Transition matrix of land use types between 2000 and 2020 (km^2^).

2000	2020					
	Cropland	Forest	Grassland	Waterbody	Construction land	Bare land
Cropland	81559	9552	38439	1335	8515	392
Forest	7883	47901	11177	199	588	173
Grassland	34262	12001	68430	625	2174	2287
Waterbody	1201	184	632	1405	252	64
Construction land	4267	216	708	93	3188	19
Bare land	465	158	2103	78	262	5878

**Table 2:** The amount of NPP transferred during the change in land use types in the MRYR from 2000 to 2020 (TgC).

2000	2020					
	Cropland	Forest	Grassland	Waterbody	Construction land	Bare land
Cropland	—	1.82	-6.98	-0.21	-0.73	-0.09
Forest	-2.20	—	-2.80	-0.04	-0.05	-0.03
Grassland	7.83	2.66	—	-0.14	-0.19	-0.28
Waterbody	0.21	0.04	0.13	—	0.02	-0.01
Construction land	1.12	0.11	0.40	-0.03	—	-0.02
Bare land	0.08	0.03	0.29	0.01	0.01	—

**Figure 5:**
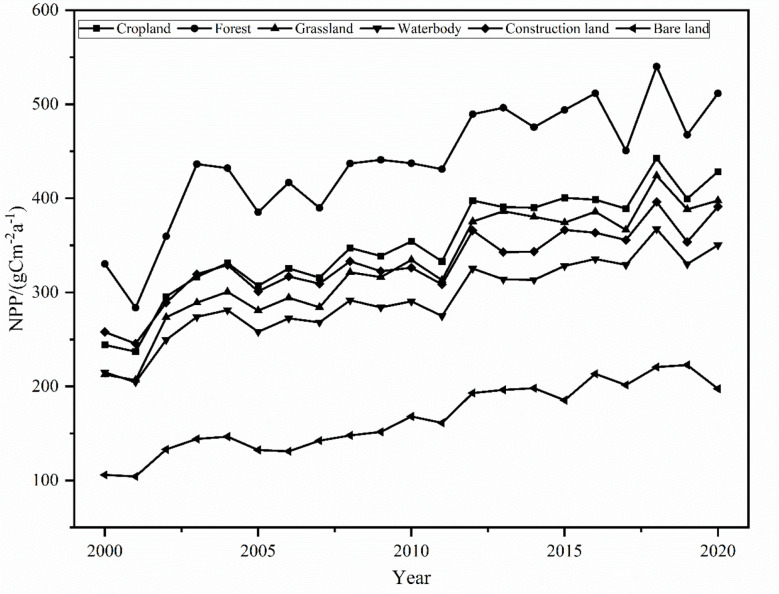
Change of NPP with different land use types in the MRYR during 2000-2020.

The annual average NPP of different land use types in the MRYR varied greatly (**Figure 5**), among which the forest NPP was always at the highest value among the six land use types, ranging from 400-500 gCm^-2^a^-1^. The second highest NPP was cropland and grassland, and the annual average NPP was 200–450 gCm^-2^a^-1^. The annual average NPP of bare land was the lowest, maintaining 104–222 gCm^-2^a^-1^. Grassland increased at a rate of 3.35 gCm^-2^a^-1^, with the most significant growth rate. Cropland increased at a rate of 3.01 gCm^-2^a^-1^, ranking second among the six land use types, and construction land increased the slowest at a rate of 2.21 gCm^-2^a^-1^. The annual average NPP of the six land use types peaked in 2018. The annual average NPP of the forest reached a maximum value of 540.14 gCm^-2^a^-1^ in 2018. The annual average NPPs of cropland and grassland were relatively close, 442.65 gCm^-2^a^-1^ and 424.12 gCm^-2^a^-1^, respectively, and the lowest NPP was observed in bare land, 220.66 gCm^-2^a^-1^, in 2018. In conclusion, the changes among different land types directly affect the spatiotemporal changes in NPP.

### 3.4 The relationship between NPP and climatic factors

**Figure 6:**
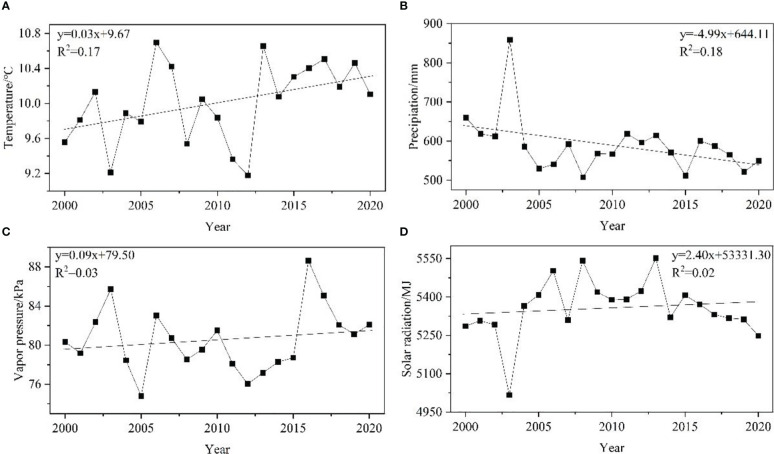
Change trends of temperature **(A)**, precipitation **(B)**, vapor pressure **(C)** and solar radiation **(D)** in the MRYR from 2000 to 2020.

**Figure 7:**
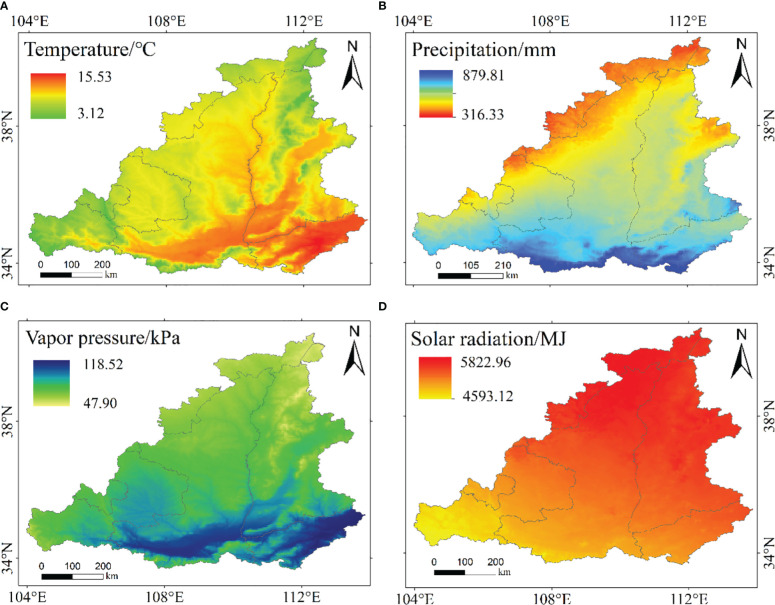
Spatial distributions of temperature **(A)**, precipitation **(B)**, vapor pressure **(C)** and solar radiation **(D)** in the MRYR from 2000 to 2020.

**Figure 8:**
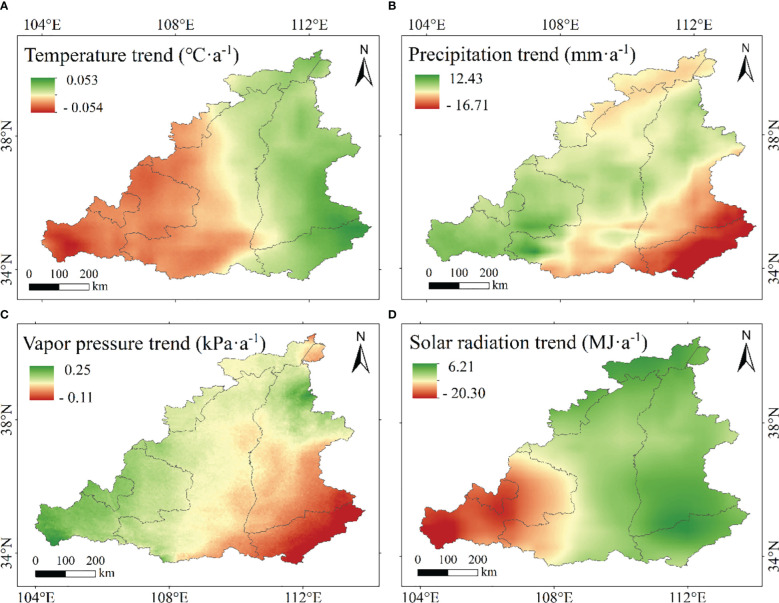
Spatial distributions of temperature **(A)**, precipitation **(B)**, vapor pressure **(C)** and solar radiation **(D)** trends in the MRYR from 2000 to 2020.

To examine the effects of meteorological factors on NPP, we analyzed the temporal and spatial distributions of four climate factors. The annual average temperature ranged from 9.4 to 10.7 °C and showed a fluctuating upward trend during the period of 2000 – 2020 (**Figure 6A**). The spatial distribution of annual average temperature in the MRYR was significantly different, showing a general trend of high temperature in the east and low temperature in the west and high temperature in the south and low temperature in the north. The temperature in the west decreased while that in the east was increased from 2000 to 2020 (**Figures 7A**, **8A**). The annual precipitation range of the MRYR was 507.5–858.6 mm. The highest precipitation occurred in 2003, and the lowest precipitation occurred in 2008. The overall trend of precipitation was downward (**Figure 6B**). The special topographic conditions in the MRYR caused obvious regional differences in the annual average rainfall, decreasing from southeast to northwest. The precipitation in the eastern part of Gansu Province, the central part of Shaanxi Province and the northern part of Shanxi Province was on the rise, while the precipitation in the western part of Henan Province and the southern part of Inner Mongolia was on the decline (**Figures 7B**, **8B**). From 2000 to 2020, the average annual water vapor pressure in the MRYR was 80.5 kPa, and the annual average water vapor pressure ranged from 74.8 to 88.6 kPa. The lowest value of water vapor pressure occurred in 2005, and the highest value occurred in 2016. There was no obvious increasing trend (**Figure 6C**). In spatial distribution, the south was spatially higher than the north. In the southeastern MRYR, the water vapor pressure showed a downward trend, while in other parts of the region, it showed an upward trend from 2000 to 2020 (**Figures 7C**, **8C**). The average solar radiation in the MRYR was 5357.73 MJ. The minimum value was 5016.27 MJ, which occurred in 2003, and the maximum value was 5551.6 MJ, which occurred in 2013 (**Figure 6D**). Overall, the average solar radiation in the MRYR had no obvious increasing trend. Spatially, it showed a gradual downward trend from north to south. The eastern part of the MRYR showed an upward trend, while the western part of the MRYR showed a downward trend from 2000 to 2020 (**Figures 7D**, **8D**).

**Figure 9:**
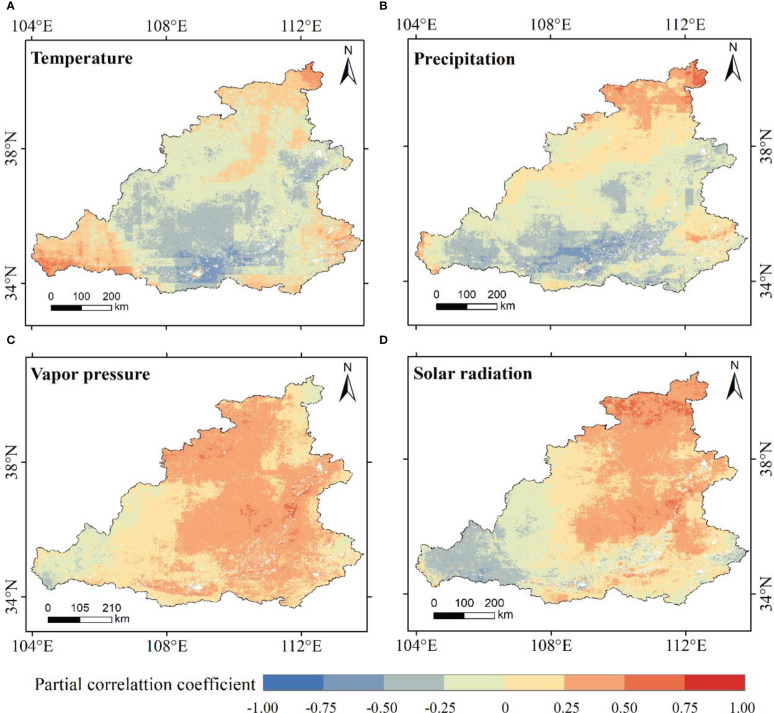
Partial correlation coefficient between NPP and temperature **(A)**, precipitation **(B)**, vapor pressure **(C)**, and solar radiation **(D)**.

**Figure 10:**
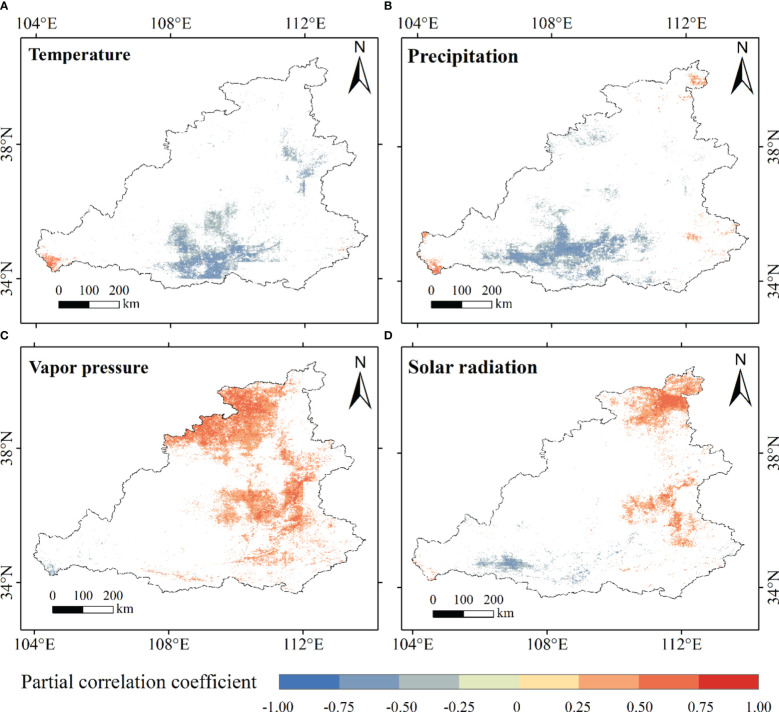
The partial correlation coefficient between NPP and temperature **(A)**, precipitation **(B)**, vapor pressure **(C)**, and solar radiation **(D)** was tested by T.

Because vegetation needs to synthesize organic matter and store energy through photosynthesis, a single climatic factor cannot fully explain this process. Therefore, the third-order partial correlation coefficient was used to study the effects of temperature, precipitation, water vapor pressure, and solar radiation on NPP in the MRYR. The partial correlation coefficient between the NPP and temperature was between -0.87–0.88 (**Figure 9A**). A total of 86.74% of the MRYR was negatively correlated with temperature. It is mainly distributed in the Loess Plateau region, where the ecological environment is fragile. The increase in air temperature increased surface evapotranspiration and restrained vegetation growth. At the spatial scale, the positive correlation region was mainly distributed in southern Gansu and western Henan, and the area of negative correlation was mainly concentrated in most of Shaanxi, Shanxi, and eastern Gansu. Only 0.089% of the negative correlation area passed the significance test of P< 0.05 and was mainly distributed in central Shaanxi (**Figure 10A**). The partial correlation coefficient between NPP and annual precipitation ranged from -0.88 to 0.85 (**Figure 9B**), and the negative correlation area accounted for 77.89%, which was distributed in most of Shaanxi, central Shanxi, and eastern Gansu. The regions with positive correlations accounted for 22.11% of the total area and were mainly distributed in southern Inner Mongolia and southeastern Shanxi. The partial relationship between NPP and precipitation showed that only 11.03% of the MRYR passed the significance test, and 9.97% of the area showed significant negative related trends, mainly distributed in central Shaanxi (**Figure 10B**). Precipitation in this area was abundant, heavy and concentrated, resulting in flood disasters and damage to vegetation. In addition, the increase in precipitation is usually accompanied by an increase in cloud cover, which prevents solar radiation from directly reaching the surface, inhibits vegetation photosynthesis, and limits the accumulation of NPP (Liu et al., 2019). The positive correlation between NPP and water vapor pressure accounted for a large proportion of the region (**Figure 9C**), at 87.60% of the total area, primarily concentrated in Ordos, Yulin City, Taiyuan City, Jinzhong City and Sanmenxia City, of which 20.43% of the areas passed the significance test of P< 0.05, mainly concentrated in northwest Shaanxi and southern Shanxi (**Figure 10C**). The negative correlation area only accounted for 12.40% of the MRYR, mainly distributed in eastern Gansu and southern Ningxia, and most areas did not pass the significance test. Vapor pressure indirectly represents the moisture content in the air and is generally positively correlated with temperature and precipitation (Xie et al., 2020). In the southern MRYR, the water vapor pressure is high, and there are suitable temperatures and precipitation for vegetation growth. The partial correlation coefficient between NPP and annual average solar radiation was-0.92 – 0.84 (**Figure 9D**), and 61.05% of the areas showed a positive correlation trend, of which 8.48% of the positive correlation areas passed the significance test of P<0.05 (**Figure 10D**). The negative correlation area accounted for 48.95% of the MRYR, and only 2.17% of the negative correlation area passed the significance test of P<0.05. The NPP at the junction of Inner Mongolia, Shanxi and Shaanxi Provinces increased significantly with increasing solar radiation. In this area, solar radiation is strong, and sufficient light stimulates vegetation photosynthesis. According to the statistical principle, the positive correlation between water vapor pressure and NPP in the MRYR was more obvious than the other three climatic factors, followed by solar radiation, while the contributions of temperature and precipitation were relatively weak.

## 4 Discussion

### 4.1 Spatiotemporal variation characteristics of NPP

During the period from 2000 to 2005, the vegetation coverage in the MRYR gradually increased, and the NPP showed a significant upward trend, with a spatial pattern of decreasing from south to north. Some scholars have studied the change trend of NPP in the Yellow River basin from 2000 to 2015 and from 2000 to 2019, indicating that the NPP increased from north to south, which is consistent with the conclusion in this paper (Wang et al., 2021; Tian et al., 2022). The lowest annual average NPP in 2001 was 198.21 gCm^-2^a^-1^. In the following three years, the NPP increased significantly. The “Grain for Green” policy was implemented in 2001, which caused a rapid decline in the agricultural planting area in the MRYR, while the newly planted forest and grass coverage were low, resulting in a decline in NPP (Xie et al., 2014). From 2005 to 2020, the NPP of the MRYR increased at a rate of 2.83 gCm^-2^a^-1^. At the spatial scale, the higher NPPs were mainly in mountain areas such as Ziwuling and Qinling, where forest and grassland are widely distributed. The natural forests were mainly composed of secondary broad-leaved forest, mixed coniferous, broad-leaved forest and temperate coniferous forest. The tree species are *Betula platyphylla*, *Populus davidiana* and *Querusmongolica*. The main species of shrubs include *Hippophae rhamnoides*, *Pinus tabuliformis* and *Spiraea salicifolia*. The common characteristics of these tree species are heliophilous, drought resistance, barren tolerance, wind and sand resistance, and strong adaptability to soil. Therefore, the NPP value in this area was high. In the northern region, the Mu Us Sandy Land had a fragile ecological environment and low vegetation coverage and the NPP was generally lower than 300 gCm^-2^a^-1^. The NPP in the central and western regions of Shanxi was low because the northeast trend of Lvliang Mountain blocked the water vapor from the east, resulting in less rainfall in this region and limiting the growth of vegetation (Tian et al., 2019).

### 4.2 Relationship between NPP and climate factors

Temperature, precipitation, and other climatic factors are the key elements that influence the growth of vegetation in the natural environment (Tripathi et al., 2019; Ren et al., 2020). At present, the dominant climatic factors that affect NPP are controversial. Temperature and precipitation were mainly selected to study the influence of climate factors on NPP. Compared with temperature, precipitation has a stronger correlation with NPP in most studies (Tian et al., 2019; Wang et al., 2021). However, some studies have shown that temperature has a stronger correlation with NPP (Guo et al., 2021; Tian et al., 2022). There were two main reasons for this difference. On the one hand, the year of study was different, and on the other hand, the method of obtaining data was different. Almost no articles have studied the correlation between water vapor pressure and NPP in the Yellow River Basin.

The influence of temperature on NPP is a complex process. On the one hand, the increase in temperature within a certain range can enhance soil microbe activity, accelerate the photosynthesis rate of vegetation, promote the accumulation of plant organic matter and is conducive to improving NPP (Keyser et al., 2000); on the other hand, very high temperatures have the potential to inhibit plant growth. An increase in temperature beyond the optimum temperature for vegetation growth will accelerate the evaporation of surface water. To avoid being unable to meet their own needs due to water shortages, plants close their stomata, reduce their photosynthetic rate and reduce the accumulation of dry matter, which is not conducive to improving NPP (Xu et al., 2020). The influence of temperature on NPP changes in the MRYR was studied in this paper. The results showed that the increase in air temperature was not conducive to the accumulation of NPP in 86.74% of the MRYR. This may be related to the fact that the increase in temperature accelerated the evaporation of soil moisture, leading to the drying of soil, the decline in the plant photosynthetic rate and the decline in NPP. This conclusion is consistent with Wang’s conclusion that the significant correlation was weak between temperature and NPP in Shaanxi Province from 2000 to 2015 (Wang et al., 2018). In the northwest MRYR, the temperature is low, and the annual precipitation is low. Deciduous coniferous forests are suitable for living in low temperature and dry areas. Therefore, deciduous coniferous forests are widely distributed in this region, and the NPP of deciduous coniferous forests was lower than that of evergreen broad-leaved forests. Therefore, the NPP in the northwestern MRYR was low (Du et al., 2022).

At present, there are relatively few research results on the inhibitory effect of precipitation on NPP, mainly on the Qinghai-Tibet Plateau (Xu et al., 2020). Precipitation is the main water source of vegetation in the MRYR, which can change soil moisture and fertility. Relevant studies have shown that with the increase in precipitation, the growing season of vegetation is shortened (Xu et al., 2020), and the precipitation in high-altitude areas can not only reduce the content of water mist in the mountains but also improve the effective solar radiation to a certain extent, which is conducive to improving the growth and development level of vegetation. The study showed that precipitation was the key factor restricting the growth of grassland vegetation. The main reason may be that water conditions severely limit the growth of grassland vegetation in warm and dry areas (Niu et al., 2008). With the increase in precipitation, the amount of clouds also increases, resulting in the reduction in solar radiation required for photosynthesis and inhibiting the increase in vegetation productivity (Liang et al., 2015). In 2011, the precipitation in the MRYR increased, while the NPP decreased, mainly because the precipitation in the MRYR was mainly concentrated in summer. The lower part of the terrain was prone to flooding. The soil was anoxic, and the plants did not breathe oxygen and produced excessive alcohol and lactic acid, leading to the death of plants. Therefore, precipitation limits the increase in NPP in the MRYR. The partial correlation analysis of NPP and precipitation in the MRYR showed that the increase in rainfall in most of Shaanxi, central Shanxi, and eastern Gansu Province inhibited the accumulation of NPP. High precipitation in this area inhibited vegetation growth.

When the moisture content in the air is sufficient, the water vapor pressure is higher, which is conducive to soil moisture retention and promotes vegetation to absorb the water and nutrients needed for growth. The vegetation in the MRYR with sufficient water vapor had better growth and higher NPP, and the area where the positive effect of water vapor pressure on NPP accounted for 87.60%, indicating that water vapor pressure was a key climatic factor affecting NPP in the MRYR. Scholars have studied the climatic factors affecting vegetation growth in the Altay region of Xinjiang and found that water vapor pressure was beneficial to the accumulation of NPP, which was similar to the results of this study (Huang et al., 2022).

The photosynthesis of vegetation cannot be separated from solar radiation, which is the source of the ability of vegetation growth, promotes the synthesis of chlorophyll in plant cells, and causes vegetation to accumulate organic matter. There was a significant positive correlation between solar radiation and NPP, mainly distributed in the northern MRYR, where the intensity of solar radiation was high, which promoted vegetation photosynthesis, and the promoting effect of enhanced radiation exceeded the inhibiting effect of drought. In the Lvliang Mountain areas, there is relatively little disturbance from human activities. Dominant tree species such as *Hippophae rhamnoides*, *Caragana korshinskii* and *Pinus tabulaeformis* were densely distributed, and these tree species were heliophilous. In these areas, the sunshine time was long, so vegetation grew vigorously and promoted the increase in NPP. This scholar studied the spatial and temporal changes in vegetation in Hunan Province and showed that solar radiation promoted the increase in NPP, which was similar to the conclusion that NPP was mainly affected by solar radiation in the MRYR obtained in this study (Yan et al., 2022). The reason was that the enhanced solar radiation made vegetation accept more light, photosynthesis was strengthened, and the accumulation of dry matter in vegetation was increased. The study was found that the effect of solar radiation on different vegetation types were different, and the effect of solar radiation on forests was stronger than that on precipitation and temperature (Du et al., 2022). In addition, it has been found that NPP is negatively correlated with solar radiation in most areas of the Loess Plateau (Xie et al., 2014), which was not consistent with the conclusion that NPP was mainly affected by solar radiation obtained in this paper. Most likely because the study was conducted at different scales, with different vegetation types and periods, the driving mechanisms of climate factors on NPP were also different.

### 4.3 Impact of land use change on NPP

The rapid change in land use on the space-time scale has changed the surface environmental elements, resulting in a change in the energy flow and material circulation function of the earth system, which had a great impact on the accumulation of vegetation organic matter and then affected NPP (Imhoff et al., 2004). From 2000 to 2020, the significant increase in NPP in forest and grassland was mainly related to the planting area and dense vegetation growth. The cropland in the MRYR decreased by 10156 km^2^ and was mainly converted into forest and grassland. The difference in crop types and the rise in yield were the key factors influencing the cropland NPP increase. Through continuous practice, the MRYR has gradually reduced the types of crops with large water demand, such as corn. Instead, the planting area of drought-resistant crops was expanded, such as soybean, sweet potato and wheat. In the hilly areas where there was little rain and large temperature difference, the apples, pears and walnuts were planted, and high-quality pasture was actively developed to promote the steady increase in NPP (Chen et al., 2005; Zhou et al., 2021; Huang et al., 2022). The photosynthesis of phytoplankton was influenced by light intensity, which raised NPP in the water. According to incomplete statistics, there are more than 200 species of phytoplankton in Sanmenxia Reservoir and Xiaolangdi Reservoir in the MRYR (Yuan et al., 2009; Han et al., 2013). Enhanced solar radiation triggered an increase in photosynthesis and NPP. The bare land included wild grassland where there were scarce plants, increasing NPP as a consequence of climate change and human activities. Since 2000, the Grain for Green Project has been implemented, some seriously degraded wasteland has been restored, and the surrounding ecological environment has improved. The construction of the reservoir has improved the surrounding ecological environment reducing soil erosion and promoting ecological restoration in this region. The vegetation of bare land recovered significantly and promoted the increase in NPP (Liu et al., 2022). Construction land increased by 6488 km^2^, mainly from cropland and bare land, resulting in a reduction in NPP. From 2000–2020, on the one hand, with the national economy of China expanding quickly, a large number of residents migrated to cities, there was accelerated expansion of Taiyuan and Xi’an, and there was rapid transformation of forest and grassland with higher vegetation productivity into land with lower productivity for construction, which led to a decrease in NPP (Milesi et al., 2003). On the other hand, the large-scale migration of the mountain population to cities and towns promoted the restoration of mountain vegetation, which led to an increase in the NPP of mountain vegetation (Wang and Li, 2018). In addition, with the gradually significant effect of ecological policies such as “Grain for Green”, cropland with low NPP was transformed into forest and grassland with high NPP, which promoted the growth of NPP (Wang et al., 2018). Overall, the increase in forest and grassland in the MRYR promoted the increase in NPP. Therefore, reasonable planning of different land use types was of great significance to the accumulation of regional NPP.

### 4.4 Uncertainty

Although this study provided a comprehensive analysis of NPP changes and climate effects in the MRYR, there may still be some limitations. The MRYR encompasses a wide area and has a complex natural environment, so it is difficult to measure NPP data in the field. Therefore, the MOD17A3HGF data were adopted to study NPP in the MRYR and lacked a comparison of field measurement data. Climate data were extracted from the GEE cloud platform ERA5 reanalysis dataset and TerraClimate dataset. Due to the spatial resolution and parameter setting of remote sensing images (Decuyper et al., 2020; Shen et al., 2020; Li et al., 2022), the climate change in the MRYR from 2000 to 2020 cannot be completely and accurately simulated. However, it does not affect the correctness of the conclusions of this study on the spatiotemporal dynamic changes in NPP and its influencing factors in the MRYR. However, data products with higher accuracy should be sought in the future. This paper mainly analyzed the impacts of four climate factors and land use type changes on the spatiotemporal changes in NPP in the MRYR. The partial correlation between NPP and four climatic factors in some regions did not pass the significance test, indicating that the influence of climate on NPP is greatly reduced due to the intervention of human activities. Human activities such as accelerated urbanization processes, construction land occupation of forestland, and unreasonable farming have damaged the ecological environment of the MRYR (Niu et al., 2021), resulting in vegetation degradation having a certain extent of impact on NPP. Otherwise, the factors affecting NPP are not only temperature, precipitation, solar radiation and water vapor pressure but also other factors that also influence NPP by affecting vegetation growth, such as soil moisture, atmospheric CO_2_ concentration and cloud cover. Therefore, it is necessary to further analyze the driving mechanism of NPP from multiple perspectives to more comprehensively understand ecosystem changes under the background of climate change in the future.

## 5 Conclusions

From 2000 to 2020, the NPP in the MRYR showed a significant upward trend, with an annual average of 319.24 gC·m^-2^a^-1^. The NPP in the southern MRYR was higher than that in the northern MRYR, showing obvious latitudinal zonality. A total of 87.68% of the study regions showed a significant increasing trend. The land use types were mainly cropland, forest and grassland. The implementation of the policy of “Grain for Green” policy reduced the area of cropland the most, which was mainly converted to forest and grassland. The average NPP of different land use types changed at different rates and showed a trend of fluctuating growth. The amount of forestland NPP was the highest, with a shift to other land-use types leading to a reduction in NPP. The conversion of grassland to forest and cropland had a significant influence on the NPP increase. The transformation of construction land, waterbodies and bare land had little effect on NPP. Temperature was negatively correlated with NPP in most parts of the MRYR and water vapor pressure was positively related to the northwest of localized NPP increase. In the MRYR, initial results were achieved through a series of ecological restoration measures. The conversion of cropland into forest led to the largest increase in NPP, but the change in land cover type dominated by urban expansion led to a decline in NPP and carbon sequestration capacity of vegetation. Therefore, under the background of high-quality development and climate change in the future, forest coverage should be increased in humid region, grassland vegetation types should be enriched in semi-arid region, high-quality farmland should be protected in humid and sub-humid region, urban garden layout should be optimized in cities, the remediation of unused land should be focused on in the MRYR. Thus, land use pattern would be reasonably optimized to improve the carbon sequestration capacity of regional vegetation.

## Data availability statement

Publicly available datasets were analyzed in this study. This data can be found here: NPP data were derived from the MOD17A3HGF of NASA (https://ladsweb.modaps.eosdis.nasa.gov/) DEM data were derived from the geospatial data cloud (https://www.gscloud.cn/), which mainly extracted the boundary of the MRYR. The land use data were extracted according to the basin boundary from the resource and environmental science and data center (https://www.resdc.cn/). Meteorological data were obtained from Terra Climate University of Idaho’s monthly climate and climate water balance dataset on global land surfaces (IDAHO_EPSCOR/TERRACLIMATE).

## Author contributions

WX participated in data analysis and original draft writing, and polished the manuscript. LR contributed to writing and supervising the study. All authors contributed to the article and approved the submitted version.
